# Incidence of pre‐eclampsia in triplet pregnancies with and without embryo reduction

**DOI:** 10.1002/uog.70341

**Published:** 2026-09-19

**Authors:** G. Yerlikaya‐Schatten, A. Wright, S. Kristensen, O. B. Petersen, P. Chaveeva, A. J. Vivanti, N. Persico, F. S. Molina, L. Lewi, E. Litwinska, K. H. Nicolaides

**Affiliations:** ^1^ Department of Obstetrics and Gynecology, Division of Obstetrics and Feto‐Maternal Medicine Medical University of Vienna Vienna Austria; ^2^ Institute of Health Research University of Exeter Exeter UK; ^3^ Centre for Fetal Medicine, Pregnancy and Ultrasound, Department of Gynecology, Fertility and Obstetrics Copenhagen University Hospital Rigshospitalet Copenhagen Denmark; ^4^ Department of Cellular and Molecular Medicine, Faculty of Health and Medical Sciences University of Copenhagen Copenhagen Denmark; ^5^ Department of Clinical Medicine, Faculty of Health and Medical Sciences University of Copenhagen Copenhagen Denmark; ^6^ University Hospital of Obstetrics and Gynecology ‘Maichin dom’ Sofia Bulgaria; ^7^ Fetal Medicine Unit, Dr. Shterev Hospital Sofia Bulgaria; ^8^ Department of Obstetrics and Gynecology, DMU Santé des Femmes et des Nouveau‐nés, Hôpital Antoine Béclère Paris Saclay University, AP‐HP Clamart France; ^9^ Fondazione IRCCS ca' Granda Ospedale Maggiore Policlinico and University of Milan Milan Italy; ^10^ Department of Obstetrics and Gynecology Hospital Universitario Clínico San Cecilio Granada Spain; ^11^ Instituto de Investigación Biosanitaria Ibs.GRANADA Granada Spain; ^12^ Department of Obstetrics and Gynecology University Hospitals Leuven Leuven Belgium; ^13^ Department of Development and Regeneration KU Leuven Leuven Belgium; ^14^ First Department of Obstetrics and Gynaecology Medical University of Warsaw Warsaw Poland; ^15^ Fetal Medicine Research Institute, King's College Hospital London UK

**Keywords:** embryo reduction, pre‐eclampsia, triplet pregnancy

## Abstract

**Objective:**

To determine the risk of pre‐eclampsia (PE) in triplet pregnancies, with and without embryo reduction, compared to singleton and twin pregnancies.

**Methods:**

This was a retrospective multicenter study of triplet pregnancies with three live, phenotypically normal fetuses at the 11–13‐week scan and a gestational age at delivery of ≥ 24 weeks. The competing‐risks model was used to determine the distribution of gestational age at delivery with PE in each type of triplet pregnancy, which was compared to datasets from 1169 twin pregnancies and 61 174 singleton pregnancies from previous cohorts. The relative risks of PE with delivery < 37, < 34 and < 32 weeks' gestation for non‐reduced and reduced triplet pregnancies, in comparison with twin and singleton pregnancies, were determined.

**Results:**

Overall, 1020 triplet pregnancies were included, of which 456 had expectant management, 468 were reduced to twin pregnancies and 96 were reduced to singleton pregnancies. The incidence of PE with delivery < 37 weeks' gestation was 9.9% for non‐reduced triplet pregnancies, 9.8% for triplet pregnancies reduced to twins, 2.1% for triplet pregnancies reduced to singleton, 7.0% for twin pregnancies and 0.8% for singleton pregnancies. The incidence of PE with delivery < 34 weeks was 7.2% for non‐reduced triplets, 4.1% for triplets reduced to twins, 0% for triplets reduced to singleton, 0.9% for twins and 0.4% for singletons. In comparison with singleton pregnancies, for non‐reduced triplets, the relative risks for PE with delivery < 37 and < 34 weeks were 12.3 (95% CI, 9.1–16.3) and 20.6 (95% CI, 14.4–29.2), respectively. The respective values for triplet pregnancies reduced to twins were 12.2 (95% CI, 9.1–16.2) and 11.6 (95% CI, 7.3–18.2), and for triplet pregnancies reduced to singleton they were 2.6 (95% CI, 0.7–9.1) and 0.0 (95% CI, 0.0–11.0). In comparison with twin pregnancies, for non‐reduced triplets the relative risks for PE with delivery < 37 and < 34 weeks were 1.4 (95% CI, 1.0–2.0) and 7.7 (95% CI, 4.0–14.9), respectively. The respective values for triplets reduced to twins were 1.4 (95% CI, 1.0–2.0) and 4.3 (95% CI, 2.1–8.9). For a given set of maternal characteristics and medical history, a non‐reduced triplet pregnancy was estimated to deliver with PE, on average, 14.5 weeks earlier than would a singleton pregnancy. Triplet pregnancies reduced to twins were estimated to deliver with PE 12.7 weeks earlier, and those reduced to singleton 6.5 weeks earlier, than would comparable singleton pregnancies.

**Conclusion:**

Non‐reduced triplet pregnancies have a 21‐fold and 8‐fold higher risk of delivery with PE at < 34 weeks' gestation compared with singleton and twin pregnancies, respectively. In triplet pregnancies reduced to twins, the relative risk of PE < 34 weeks is 4‐fold higher than that in twin pregnancies, whereas in triplet pregnancies reduced to singleton, the risk is similar to that of singleton pregnancies. © 2026 The Author(s). *Ultrasound in Obstetrics & Gynecology* published by John Wiley & Sons Ltd on behalf of International Society of Ultrasound in Obstetrics and Gynecology.

## INTRODUCTION

Pre‐eclampsia (PE) is a leading global cause of maternal and perinatal mortality and morbidity^1^. In singleton pregnancies, the incidence of PE is 3–5%, and in twin pregnancies, the risk is three times higher for overall PE and nine times higher for preterm PE (PE with delivery < 37 weeks' gestation)^2^. There is a sparsity of data for PE in triplet pregnancies.

The best method for assessing the risk of development of PE is the competing‐risks model^3^. This model allows for the estimation of patient‐specific risks of PE with delivery before any specified gestational age, utilizing maternal demographic characteristics and medical history as well as biomarkers obtained either individually or in combination at any stage during pregnancy. In the competing‐risks approach, every woman has a personalized distribution of gestational age at delivery with PE; whether or not the woman experiences PE before a specified gestational age depends on competing risks between the development of PE and delivery before PE develops. The personalized distribution comes from the application of Bayes' theorem to combine a previous distribution, which is determined from maternal factors, with likelihoods derived from biomarkers. The competing‐risks model has allowed direct comparison of the risk of PE in singleton and twin pregnancies by quantifying both the incidence and timing of delivery with the disease^4^, ^5^.

The objectives of this study were to use the competing‐risks model to estimate the risk of PE in triplet pregnancies in comparison with singleton and twin pregnancies, and to examine the extent to which embryo reduction in triplet pregnancies modifies this risk of PE.

## METHODS

### Study population

This was a retrospective multicenter study of triplet pregnancies with three live, phenotypically normal fetuses at the 11–13‐week scan and a gestational age at delivery of ≥ 24 weeks. Contributing hospitals were: Medical University of Vienna (Vienna, Austria); Copenhagen University Hospital Rigshospitalet (Copenhagen, Denmark); Dr. Shterev Hospital (Sofia, Bulgaria); Antoine Béclère Hospital (Clamart, France); University of Milan (Milan, Italy); Hospital Universitario Clínico San Cecilio (Granada, Spain); University Hospitals Leuven (Leuven, Belgium); and Medical University of Warsaw (Warsaw, Poland). Findings in our cohort of triplet pregnancies were compared to those of 1169 twin pregnancies that were included in the EVENTS trial, which examined the effect of early vaginal progesterone for the prevention of spontaneous preterm birth in twins^6^, and 61 174 singleton pregnancies included in a non‐interventional study on first‐trimester screening for PE^7^.

All women with a singleton, twin or triplet pregnancy attended a routine clinical visit at 11 + 0 to 13 + 6 weeks' gestation, which included recording of maternal characteristics and medical history, and ultrasound examination to determine the gestational age using the crown–rump length (the longest fetus was considered in twin and triplet pregnancies) and chorionicity from the lambda sign in multiple pregnancies, and for the diagnosis/exclusion of major abnormalities^8^, ^9^. We included pregnancies with phenotypically normal fetuses at the first‐trimester scan that ended in live birth or stillbirth of a phenotypically normal neonate at ≥ 24 weeks' gestation. We excluded pregnancies with aneuploidy or major fetal abnormality and those ending in termination, miscarriage or fetal death < 24 weeks.

### Outcome measures

The outcome measures were the incidence of PE with delivery < 37, < 34 and < 32 weeks' gestation, in non‐reduced and reduced triplet pregnancies, and the relative risks (RRs) of PE in comparison to singleton and twin pregnancies.

Data on pregnancy outcome were collected from the hospital maternity records of the women and PE was defined, as per the American College of Obstetricians and Gynecologists (ACOG) guidelines, as chronic or gestational hypertension with development of at least one of the following: new‐onset proteinuria (≥ 300 mg protein in a 24‐h urine collection or a protein‐to‐creatinine ratio ≥ 0.3), serum creatinine > 97 μmol/L in the absence of underlying renal disease, serum transaminases more than twice the limit of normal, platelet count < 100 000/μL, persistent headaches or visual symptoms, or pulmonary edema^10^. Chronic hypertension was defined as systolic blood pressure (BP) ≥ 140 mmHg and/or diastolic BP ≥ 90 mmHg, on at least two occasions, 4 h apart, documented before pregnancy or < 20 weeks^11^. Gestational hypertension was defined as new‐onset hypertension at ≥ 20 weeks in a previously normotensive woman^10^.

### Statistical analysis

Data are summarized as mean and 95% CI for continuous variables and *n* (%) with 95% CI for categorical variables. As an exploratory analysis, the incidence of PE with delivery < 32, < 34 and < 37 weeks in non‐reduced triplets, triplets reduced to twins, triplets reduced to singletons, and twins was compared to that in singletons, and the incidences in non‐reduced triplets, triplets reduced to twins and triplets reduced to singletons were compared to those in twins, by estimating crude RRs.

We applied the Fetal Medicine Foundation (FMF) competing‐risks model, developed for singleton pregnancies, which incorporates maternal characteristics and medical history to define the mean of a Gaussian distribution for gestational age at delivery with PE^3^. This model was used to calculate an expected singleton mean gestational age at delivery with PE for each triplet pregnancy. Subsequently, a Gaussian parametric survival model for gestational age at delivery with PE was fitted for triplet pregnancies. The predicted singleton mean gestational age was included as an offset, and indicators for non‐reduced triplets, reduction to twins and reduction to singleton were included as covariates. Model results were summarized both as estimated effects, expressed in weeks relative to the expected singleton mean gestational age at delivery with PE, and graphically. Graphical summaries included plots of the fitted distributions of gestational age at delivery with PE, as well as plots of the cumulative incidence of delivery with PE.

The statistical software package RStudio (Posit PBC, Boston, MA, USA) was used for data analysis. The package survival was used for survival analysis and the package PropCIs was used for calculation of confidence intervals for proportions.

## RESULTS

Overall, 1020 triplet pregnancies were included. These comprised 158 pregnancies from Medical University of Vienna, 377 from Copenhagen University Hospital Rigshospitalet, 126 from Dr. Shterev Hospital, 121 from Antoine Béclère Hospital, 92 from University of Milan, 72 from Hospital Universitario Clínico San Cecilio, 37 from University Hospitals Leuven and 37 from Medical University of Warsaw. Table 1 reports the maternal and pregnancy characteristics of the triplet pregnancies in our cohort, stratified by reduction type, and of the twin^6^ and singleton^7^ pregnancies from previous cohorts. Of the triplet pregnancies, 456 underwent expectant management, 468 underwent embryo reduction to a twin pregnancy and 96 underwent embryo reduction to a singleton pregnancy. The incidence of PE with delivery < 37 weeks' gestation was 9.9% for non‐reduced triplet pregnancies, 9.8% for triplets reduced to twins, 2.1% for triplets reduced to singleton, 7.0% for twin pregnancies and 0.8% for singleton pregnancies. The incidence of PE with delivery < 34 weeks was 7.2% for non‐reduced triplets, 4.1% for triplets reduced to twins, 0% for triplets reduced to singletons, 0.9% for twins and 0.4% for singletons, and the incidence of PE with delivery < 32 weeks was 3.1%, 1.5% and 0% for non‐reduced triplets, triplets reduced to twins and triplets reduced to singleton, respectively, 0.6% for twins and 0.2% for singletons.

**Table 1:** Maternal and pregnancy characteristics of study population of triplet, twin and singleton pregnancies

	Triplet pregnancies				
Characteristic	Non‐reduced (*n* = 456)	Reduced to twins (*n* = 468)	Reduced to singleton (*n* = 96)	Twin pregnancies^6^ (*n* = 1169)	Singleton pregnancies^7^ (*n* = 61 174)
Maternal age (years)	32.5 (32.0–33.1)	33.1 (32.6–33.6)	34.8 (33.7–36.0)	33.9 (33.6–34.2)	31.0 (30.9–31.0)
Maternal weight (kg)	69.2 (67.8–70.5)	66.3 (64.9–67.7)	67.5 (64.5–70.5)	70.1 (69.2–70.9)	69.9 (69.8–70.0)
Maternal height (cm)	165.6 (164.7–166.4)	166.2 (165.1–167.2)	167.6 (166.3–168.9)	165.4 (165.1–165.8)	164.5 (164.4–164.5)
Maternal BMI (kg/m^2^)	25.8 (24.2–27.3)	26.3 (23.0–29.7)	23.9 (23.0–24.9)	25.6 (25.3–25.9)	25.8 (25.8–25.9)
GA at first‐trimester screening (days)	87.3 (86.9–87.8)	83.3 (82.9–83.7)	84.0 (83.1–85.0)	92.2 (91.9‐92.4)	89.2 (89.1–89.2)
Maternal ethnicity					
White	432 (94.7) [92.3–96.6]	458 (97.9) [96.1–99.0]	94 (97.9) [92.7–99.7]	965 (82.5) [80.2–84.7]	44 684 (73.0) [72.7–73.4]
Black	15 (3.3) [1.9–5.4]	2 (0.4) [0.1–1.5]	1 (1.0) [0–5.7]	128 (10.9) [9.2–12.9]	10 108 (16.5) [16.2–16.8]
South Asian	4 (0.9) [0.2–2.2]	5 (1.1) [0.3–2.5]	1 (1.0) [0–5.7]	46 (3.9) [2.9–5.2]	3446 (5.6) [5.5–5.8]
East Asian	3 (0.7) [0.1–1.9]	3 (0.6) [0.1–1.9]	0 (0) [0–3.8]	11 (0.9) [0.5–1.7]	1407 (2.3) [2.2–2.4]
Mixed	2 (0.4) 0.1–1.6]	0 (0) [0–0.8]	0 (0) [0–3.8]	19 (1.6) [1.0–2.5]	1529 (2.5) [2.4–2.6]
Medical history					
Chronic hypertension	4 (0.9) [0.2–2.2]	1 (0.2) [0–1.2]	2 (2.1) [0.3–7.3]	18 (1.5) [0.9–2.4]	798 (1.3) [1.2–1.4]
Diabetes mellitus	7 (1.5) [0.6–3.1]	2 (0.4) [0.1–1.5]	0 (0) [0–3.8]	11 (0.9) [0.5–1.6]	510 (0.8) [0.8–0.9]
SLE/APS	2 (0.4) [0.1–1.6]	2 (0.4) [0.1–1.5]	0 (0) [0–3.8]	1 (0.1) [0–0.5]	111 (0.2) [0.1–0.2]
Conception mode					
*In‐vitro* fertilization	203 (44.5) [39.9–49.2]	196 (41.9) [37.4–46.5]	48 (50.0) [39.6–60.4]	328 (28.1) [25.5–30.7]	1644 (2.7) [2.6–2.8]
Ovulation drugs	82 (18.0) [14.6–21.8]	178 (38.0) [33.6–42.6]	20 (20.8) [13.2–30.3]	79 (6.8) [5.4–8.4]	538 (0.9) [0.8–1.0]
Spontaneous	171 (37.5) [33.0–42.1]	94 (20.1) [16.5–24.0]	28 (29.2) [20.3–39.3]	762 (65.2) [62.4–67.9]	58 992 (96.4) [96.3–96.6]
Pregnancy history					
Parous, no previous PE	173 (37.9) [33.5–42.6]	202 (43.2) [38.6–47.8]	55 (57.3) [46.8–67.3]	502 (42.9) [40.1–45.8]	30 253 (49.5) [49.1–49.9]
Parous, previous PE	6 (1.3) [0.5–2.8]	4 (0.9) [0.2–2.2]	0 (0) [0–3.8]	24 (2.1) [1.3–3.0]	1846 (3.0) [2.9–3.2]
Nulliparous	277 (60.7) [56.1–65.3]	262 (56.0) [51.4–60.5]	41 (42.7) [32.7–53.2]	643 (55.0) [52.1–57.9]	29 075 (47.5) [47.1–47.9]
Chorionicity					
Monochorionic	25 (5.5) [3.6–8.0]	2 (0.4) [0.1–1.5]	0 (0) [0–3.8]	267 (22.8) [20.5–25.4]	NA
Dichorionic	157 (34.4) [30.1–39.0]	75 (16.0) [12.8–19.7]	73 (76.0) [66.3–84.2]	902 (77.2) [74.6–79.5]	NA
Trichorionic	274 (60.1) [55.4–64.6]	391 (83.5) [79.9–86.8]	23 (24.0) [15.8–33.7]	NA	NA
Pregnancy outcome					
GA at delivery (weeks)	32.6 (32.3–32.8)	35.2 (34.9–35.5)	38.4 (37.9–39.0)	35.4 (35.2–35.6)	39.6 (39.6–39.7)
PE < 37 weeks	45 (9.9) [7.3–13.0]	46 (9.8) [7.3–12.9]	2 (2.1) [0.3–7.3]	83 (7.0) [5.6–8.5]	493 (0.8) [0.7–0.9]
PE < 34 weeks	33 (7.2) [5.0–10.0]	19 (4.1) [2.5–6.3]	0 (0) [0–3.8]	11 (0.9) [0.5–1.6]	215 (0.4) [0.3–0.4]
PE < 32 weeks	14 (3.1) [1.7– 5.1]	7 (1.5) [0.6–3.1]	0 (0) [0–3.8]	7 (0.6) [0.2–1.2]	116 (0.2) [0.2–0.2]

Data are given as mean (95% CI) or *n* (%) [95% CI]. APS, antiphospholipid syndrome; BMI, body mass index; GA, gestational age; NA, not applicable; PE, pre‐eclampsia; SLE, systemic lupus erythematosus.

The RRs for delivery with PE at < 37, < 34 and < 32 weeks' gestation in non‐reduced and reduced triplet pregnancies and twin pregnancies^6^ in comparison with singleton pregnancies^7^, and in non‐reduced and reduced triplet pregnancies in comparison with twin pregnancies^6^, are shown in Table 2 and Figure 1. For non‐reduced triplets, in comparison with singleton pregnancies, the RRs for PE with delivery at < 37, < 34 and < 32 weeks were 12.3 (95% CI, 9.1–16.3), 20.6 (95% CI, 14.4–29.2) and 16.2 (95% CI, 9.4–27.7), respectively. For triplets reduced to twins, the RRs for PE with delivery at < 37, < 34 and < 32 weeks were 12.2 (95% CI, 9.1–16.2), 11.6 (95% CI, 7.3–18.2) and 7.9 (95% CI, 3.8–16.5), respectively, and for triplets reduced to singletons, the RRs were 2.6 (95% CI, 0.7–9.1), 0.0 (95% CI, 0.0–11.0) and 0.0 (95% CI, 0.0–20.3), respectively, in comparison with singleton pregnancies. In comparison with twin pregnancies, for non‐reduced triplets the RRs for PE with delivery at < 37, < 34 and < 32 weeks were 1.4 (95% CI, 1.0–2.0), 7.7 (95% CI, 4.0–14.9) and 5.1 (95% CI, 2.1–12.3), respectively. For triplets reduced to twins, the RRs for PE with delivery < 37, < 34 and < 32 weeks were 1.4 (95% CI, 1.0–2.0), 4.3 (95% CI, 2.1–8.9) and 2.5 (95% CI, 0.9–6.8), and for triplets reduced to singletons the RRs were 0.3 (95% CI, 0.1–1.0), 0.0 (95% CI, 0.0–4.1) and 0.0 (95% CI, 0.0–6.5), in comparison with twin pregnancies.

**Table 2:** Relative risk for pre‐eclampsia (PE) with delivery < 37, < 34 and < 32 weeks' gestation in non‐reduced and reduced triplet pregnancies and twin pregnancies^6^ in comparison with singleton pregnancies^7^, and in non‐reduced and reduced triplet pregnancies in comparison with twin pregnancies^6^

	Relative risk (95% CI)		
Pregnancy type	PE < 37 weeks	PE < 34 weeks	PE < 32 weeks
Comparison with singletons^7^			
Non‐reduced triplet	12.25 (9.13–16.30)	20.59 (14.42–29.19)	16.19 (9.41–27.67)
Triplet reduced to twin	12.20 (9.12–16.19)	11.55 (7.30–18.15)	7.89 (3.75–16.46)
Triplet reduced to singleton	2.59 (0.71–9.06)	0.00 (0.00–10.95)	0.00 (0.00–20.32)
Twin^6^	8.81 (7.03–11.01)	2.68 (1.48–4.84)	3.16 (1.50–6.63)
Comparison with twins^6^			
Non‐reduced triplet	1.39 (0.98–1.95)	7.69 (3.97–14.91)	5.13 (2.14–12.27)
Triplet reduced to twin	1.38 (0.98–1.95)	4.31 (2.10–8.85)	2.50 (0.92–6.78)
Triplet reduced to singleton	0.29 (0.08–1.04)	0.00 (0.00–4.13)	0.00 (0.00–6.51)

**Figure 1:**
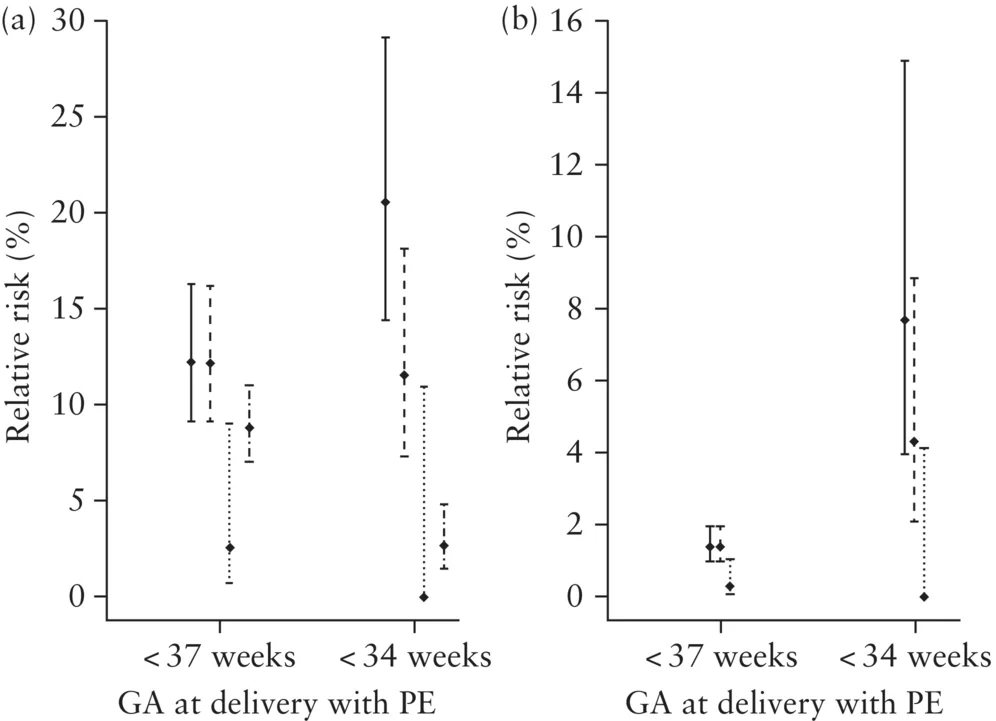
Relative risk with 95% CI for pre‐eclampsia (PE) with delivery < 37 and < 34 weeks' gestation in: (a) non‐reduced triplet pregnancies (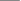), triplet pregnancies reduced to twins (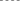), triplet pregnancies reduced to singleton (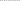), and twin pregnancies^6^ (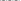), compared with singleton pregnancies^7^; and (b) non‐reduced triplet pregnancies (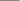), triplet pregnancies reduced to twins (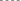) and triplet pregnancies reduced to singleton (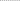), compared with twin pregnancies^6^. GA, gestational age.

Estimates of the effects of non‐reduced triplet pregnancies, triplet pregnancies reduced to twins and triplet pregnancies reduced to singleton on gestational age at delivery with PE, relative to the expected mean gestational age in singleton pregnancies^7^, are presented in Table 3. For a given set of maternal characteristics and medical history, a non‐reduced triplet pregnancy was estimated to deliver with PE, on average, 14.5 weeks earlier than would a comparable singleton pregnancy. Triplet pregnancies reduced to twins were estimated to deliver with PE 12.7 weeks earlier, and those reduced to singleton 6.5 weeks earlier, than would comparable singleton pregnancies. These shifts in mean gestational age at delivery with PE are illustrated in Figure 2.

**Table 3:** Estimates of the effects on gestational age (GA) at delivery with pre‐eclampsia of non‐reduced triplet pregnancies, triplet pregnancies reduced to twin and triplet pregnancies reduced to singleton

Pregnancy type	Estimated difference (95% CI) (weeks)	*P*
Non‐reduced triplet	−14.5 (−15.2 to −13.7)	< 0.0001
Triplet reduced to twin	−12.7 (−13.4 to −12.0)	< 0.0001
Triplet reduced to singleton	−6.5 (−9.1 to −4.0)	< 0.0001

Estimates are relative to expected singleton mean GA^7^, and are based on a hypothetical 35‐year‐old nulliparous woman of White ethnicity, 69 kg weight and 164 cm height, with no history of chronic hypertension or diabetes mellitus, reflecting a reference profile of average maternal characteristics and medical history.

**Figure 2:**
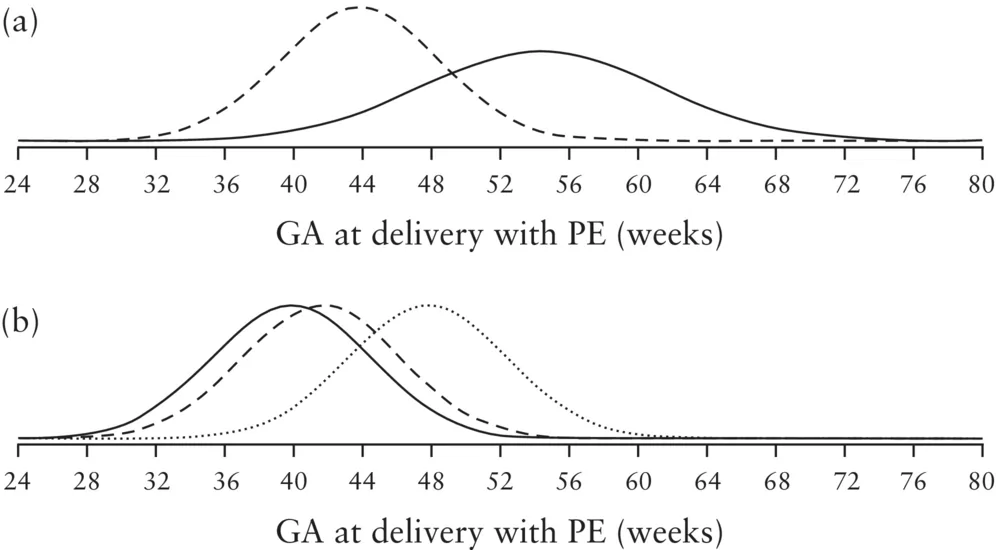
Distribution of gestational age (GA) at delivery with PE in: (a) singleton pregnancies (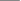) and twin pregnancies (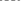); and (b) triplet pregnancies reduced to singleton (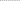), triplet pregnancies reduced to twins (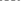) and non‐reduced triplet pregnancies (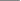). All estimates apply to a hypothetical 35‐year‐old nulliparous woman of White ethnicity, 69 kg weight and 164 cm height, with no history of chronic hypertension or diabetes mellitus, reflecting a reference profile of average maternal characteristics and medical history.

Figure 3 demonstrates a progressive increase in the cumulative incidence of PE with increasing fetal number. Non‐reduced triplet pregnancies had the highest cumulative incidence across gestation, followed by triplet pregnancies reduced to twins and then twin pregnancies, whereas triplet pregnancies reduced to singleton showed a cumulative incidence approaching that of singleton pregnancies.

**Figure 3:**
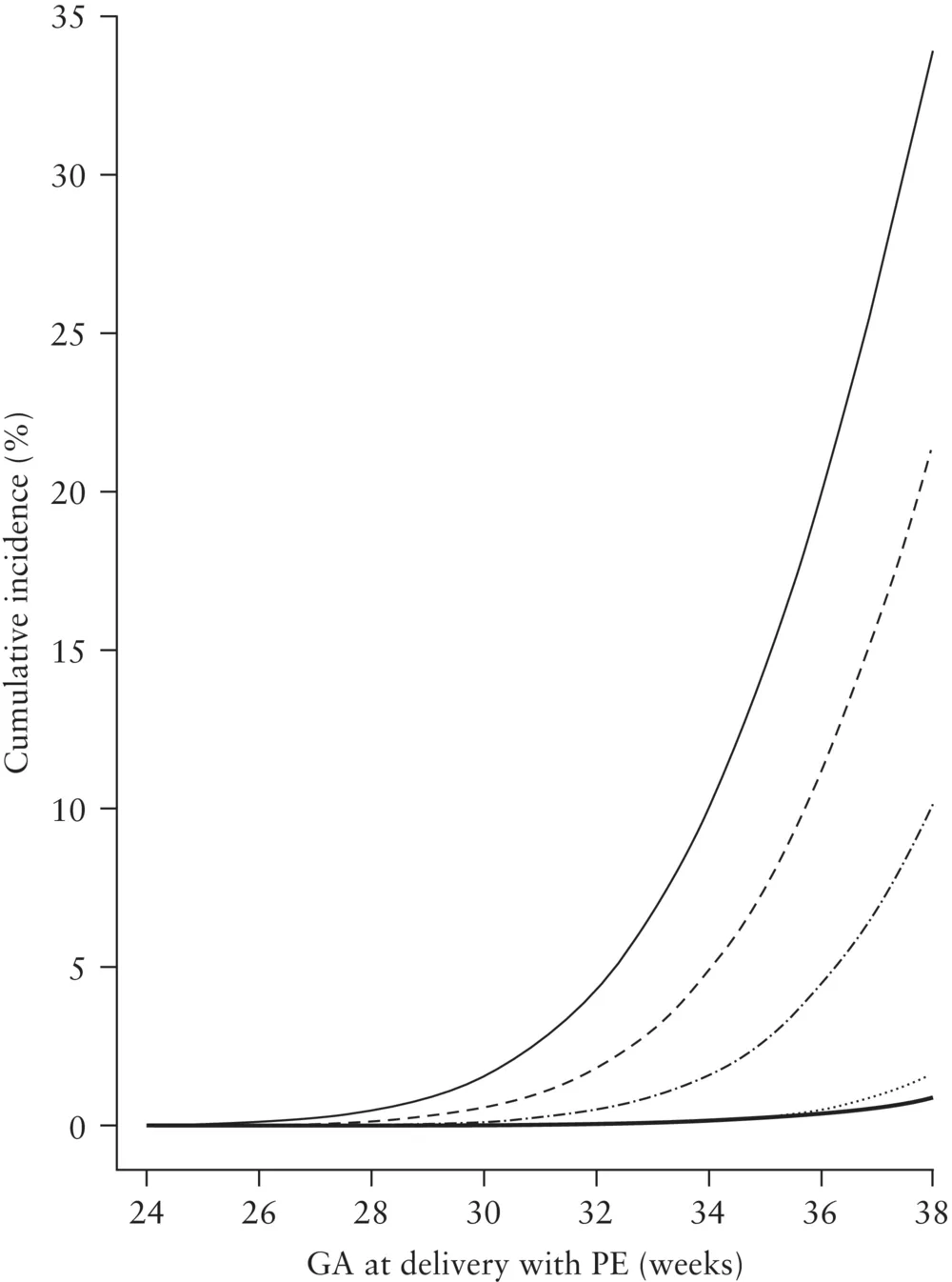
Cumulative incidence of delivery with PE, according to gestational age (GA) at delivery, in singleton pregnancies (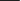), twin pregnancies (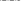), triplet pregnancies reduced to singleton (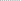), triplet pregnancies reduced to twins (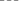) and non‐reduced triplet pregnancies (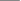). All estimates apply to a hypothetical 35‐year‐old nulliparous woman of White ethnicity, 69 kg weight and 164 cm height, with no history of chronic hypertension or diabetes mellitus, reflecting a reference profile of average maternal characteristics and medical history.

## DISCUSSION

### Main findings

This large multicenter study of triplet pregnancies, with three live, phenotypically normal fetuses at the 11–13‐week scan and a gestational age at delivery of ≥ 24 weeks, has demonstrated three main findings. First, in triplet pregnancies, the incidence of preterm PE, particularly early PE (delivery < 34 weeks' gestation), was substantially higher than that in singleton and twin pregnancies. The incidence of preterm PE was higher in non‐reduced triplet pregnancies than in twin pregnancies (9.9% *vs* 7.0%), whereas in triplet pregnancies reduced to singleton, the incidence was only 2.1%, similar to that observed in singleton pregnancies. Second, in triplet pregnancies reduced to twins, the incidence of early PE was higher than that in twin pregnancies (4.1% *vs* 0.9%). Third, in triplet pregnancies reduced to singletons, the incidence of early PE was similar to that of singleton pregnancies (0% *vs* 0.4%).

### Comparison with the literature

Our results demonstrate that the risk for development of PE is greater in pregnancies with a higher number of fetuses, but a true reflection of the increased risk should take into account that multiple pregnancies deliver earlier than singletons. These results extend previous observations in twin pregnancies, in which the rate of overall PE is three times higher than that in singleton pregnancies^2^. However, since most twin pregnancies are delivered preterm, a more relevant outcome for comparison with singleton pregnancies is preterm PE, rather than overall PE; the rate of preterm PE in twin pregnancies is nine times higher than that in singleton pregnancies^2^.

Bdolah *et al*.^12^ proposed that the increased risk of PE in twin pregnancies may be due to increased placental mass, leading to increased circulating levels of the antiangiogenic soluble fms‐like tyrosine kinase‐1 (sFlt‐1). Although no studies have reported sFlt‐1 levels in triplet pregnancies, it is likely that the levels are increased in proportion to the higher mass of placental tissue.

Several small studies have consistently reported a high rate of PE in triplet pregnancies. For example, a study of 230 triplet pregnancies reported PE or HELLP syndrome in 19% of cases^13^. Another study reported that, in 53 triplet pregnancies compared with 53 twin pregnancies matched for maternal age, parity and race, the respective rates of severe PE were 22.6% and 5.7%^14^. A study of 82 triplet pregnancies reported PE or hypertensive disorders in 46.3% of cases^15^.

The potential reversibility of PE risk after embryo reduction is another important consideration supporting the theory of placental mass. A recent systematic review and meta‐analysis showed that multifetal pregnancy reduction is associated with a reduction in hypertensive disorders of pregnancy compared with ongoing higher‐order multifetal pregnancies^16^. Nevertheless, the risk does not appear to normalize completely, suggesting that early placental and maternal cardiovascular adaptation may already be established prior to reduction. A study of 54 non‐reduced triplet pregnancies reported that the rate of PE was 30%, whereas in 59 twin pregnancies resulting from multifetal pregnancy reduction, the rate was 14%^17^. In another study, the rate of gestational hypertension was 23% in 30 triplet pregnancies reduced to twins, and it was 7% in 30 twin pregnancies^18^.

### Implications for clinical practice

In singleton pregnancies at high risk for preterm PE, prophylactic administration of low‐dose aspirin has been shown to reduce the rate of preterm PE by approximately 60%, early PE with delivery < 34 weeks by approximately 80% and PE with delivery < 32 weeks by 90%^19^, ^20^. Although the risk for preterm PE in twin pregnancies is nine times higher than that in singleton pregnancies, it is uncertain whether prophylactic aspirin would be beneficial. This is currently being investigated in a multicenter randomized controlled trial (RCT), ASPRE‐T^21^. Theoretically, a RCT could also assess the use of aspirin prophylaxis in triplet pregnancies. However, given the rarity of triplet gestations and the very large sample size that would be required to demonstrate a statistically significant reduction in PE, such a study would be difficult to conduct in practice. Nevertheless, triplet pregnancies should be considered a population at a substantially increased risk of PE, warranting close clinical surveillance for early diagnosis. If the ASPRE‐T RCT conducted in twin pregnancies finds that aspirin is beneficial, then it would be reasonable to assume that the same would be true in triplets.

### Strengths and limitations

The main strength of this study was the large number of included triplet pregnancies with three live fetuses at 11–13 weeks' gestation utilized to evaluate the incidence of PE in triplet gestations. The multicenter design enhanced the generalizability of findings. In addition, use of the competing‐risks model for prediction of gestational age at delivery with PE allowed for comparison of non‐reduced and reduced triplet pregnancies with twin and singleton pregnancies. The main limitation of the study was its retrospective design, with the potential for selection bias and unmeasured confounding.

### Conclusion

This study highlights the markedly increased risk of PE in triplet pregnancies, which remains elevated even after embryo reduction to twin pregnancy. Triplet pregnancies have a 21‐fold and 8‐fold higher risk of PE with delivery < 34 weeks' gestation compared with singleton and twin pregnancies, respectively. In triplet pregnancies reduced to twins, the RR of PE with delivery < 34 weeks is 4‐fold higher than in twin pregnancies, whereas in triplet pregnancies reduced to singleton, the risk is similar to that of singleton pregnancies.

## Data Availability

Research data are not shared.
